# Prediction of Psychosocial Sequelae in Families of Leukaemic Children

**Published:** 1988

**Authors:** Janice Culling, Richard Williams

**Affiliations:** Southmead Hospital; Bristol Children's Hospital


### A Study of the Psychological Consequences of Childhood Leukaemia in the Family: Preliminary Findings

**Janice Culling**

Southmead Hospital

**Richard Williams**

Bristol Children's Hospital

### INTRODUCTION

Medical advances during the last fifteen years have dramatically altered the course of treatment and prognosis of children diagnosed as having acute leukaemia. It is now more appropriate to consider acute leukaemia in childhood as a chronic long-term illness rather than as an acute life-threatening illness with death as the inevitable outcome.

Much of the earlier research on the psychological sequelae of childhood cancer has focussed on two main areas, namely the effects on the parents, and the effects on the afflicted child. The main emphasis of these earlier studies tended towards a mental health approach or towards examination of specific behavioural characteristics of the child and siblings. The work of Comaroff (1981) describes the emotional impact on parents on receiving the information that their child has cancer and outlines some of the conflicts faced by the family through the course of the illness. The diagnosis is generally experienced as a severe shock, the parents regarding the disease as inevitably fatal. The shock is often followed by feelings of guilt, stemming from the knowledge that they, the parents, delayed seeking help because the presenting symptoms appeared trivial. Guilt may be associated with anger, often projected onto medical personnel who in some way are perceived as having failed to make the diagnosis early enough. Having recovered from the stage of initial shock and having developed an understanding of the illness and its treatment and progress, the parents have to face months of uncertainty, never knowing whether their child will live or die.

The treatment itself is demanding and time-consuming. Special arrangements have to be made for siblings, and the parents may lose time from work and suffer financially as a result. The family may find themselves in a state of social isolation, for the stigma of having a leukaemic child may drive away the support of family and friends. The parents may have difficulty balancing the needs of the whole family against the needs of the sick child, or in maintaining appropriate standards of discipline and expectations of behaviour of the sick child. Many parents cope with and survive these conflicts while others fail, in which case psychological dysfunction may arise.

Maguire (1983) studied the psychological and social adjustment of the parents of sixty children following the diagnosis of acute leukaemia. He found that thirty per cent of mothers were suffering from an anxiety state compared with only five per cent of the mothers of children in a control group suffering benign disease. Over a third of mothers also suffered from symptoms of depression compared with only nine per cent in the control group. At twelve to eighteen months following diagnosis, a quarter of the mothers were still found to be suffering from an anxiety state or depressive illness compared with only eight per cent of the mothers in the control group. Other studies have confirmed these findings, both in Great Britain and abroad.

Several studies have focussed on the behavioural characteristics of both the siblings and the afflicted child. Maguire (1983) studying siblings using both a school-based behavioural questionnaire and parents' reports found that a third of the siblings had developed mild, moderate or severe behavioural problems during the follow-up period of twelve to eighteen months compared with only five per cent of siblings in the control group. In the case of the afflicted child, Maguire found that thirty-eight per cent of children developed behavioural problems in the follow-up period compared with only eight per cent of the children in the control group. The most common problems found included clinging dependent behaviour, temper tantrums, and a reluctance to sleep alone. There appeared to be a strong correlation between these behavioural problems and changes in the parents' behaviour towards the child. Over half the parents admitted to a considerable change in their attitudes and behaviour towards the children. Principal among these was a lessening of discipline, over-indulgence and over-protection.

### THE BRISTOL STUDY

On reviewing the research literature it is apparent that although much is known about the impact of leukaemia on families, relatively little is known about how these problems can be identified. Maguire's study showed that only one in six of the mothers who developed psychological problems disclosed these to anyone involved in their care. The focus of our own study was two-fold: firstly to look at the family from the systemic, interactional point of view and secondly to see if it would be possible to predict which families are most at risk of suffering serious psychological dysfunction. The ability to make this prediction could have important benefits in terms of offering early intervention to assist these families with their adjustment rather than wait for a crisis to occur or for the family to break down. This brief paper will set out to describe some aspects of the study and some of our preliminary findings. No attempt will be made to draw definitive conclusions as the study is at the present time incomplete.

Our study involves fourteen families followed up over a total period of three years. Families were selected on the criteria that the child had acute lymphoplastic leukaemia and was referred to the Oncology Department at Bristol Children's Hospital for the induction stage of the treatment programme. Families living outside the Avon/Somerset county boundary were excluded because of the difficulty of follow-up. The families were interviewed on two separate occasions, the first interview taking place eight weeks after initial diagnosis and the second interview one year later. The interview took the form of a semi-structured questionnaire with both parents, covering aspects of the parents' emotional and physical health, systems of family support and interaction. Information from this interview, together with information obtained from nursing and medical staff enabled the interviewer to make an assessment of the family's adjustment and functioning and to complete the Family Adjustment Scale devised by Spinetta (1981). Parents were also asked to complete behavioural questionnaires (Rutter A) on each child in the family. Each member of the family including siblings as appropriate, was asked to complete the Moos Family Environment Scale, devised to obtain a profile of family interaction and functioning. Finally each member of the family was asked to complete a Kinetic Family Drawing (KFD), an age-independent, non-verbal measure of family interaction and emotional tone, previously described by Spinetta (1981) in his study of leukaemic children and their families. The teachers of identified patients andeach sibling completed behavioural questionnaires (Rutter B).

The results of the Rutter A, and Rutter B behaviour checklist questionnaires on 11 siblings are presented in Table 1. Scores A and A1 represent parents' assessment eight weeks following diagnosis, and at one-year follow up respectively. Scores B and B1 represent teachers assessment at eight weeks following diagnosis, and at one-year follow up, respectively. A total score of 13 on the Rutter A, and a total score of 9 on the Rutter B is the criteria for significant behaviour disturbance. For the purpose of this study scores of 10 in the Rutter A and 6 in the Rutter B are taken to represent an indication of moderate behavioural disturbance.

**Table 1**

| SIB | A | A1 | B | B1 |
|---|---|---|---|---|
| 1 | 9 | 12 | 4 | 0 |
| 2 | 6 | 3 | 5 | — |
| 3 | 4 | 1 | 4 | 0 |
| 4 | 2 | 2 | 0 | 0 |
| 5 | 4 | 6 | 4 | 1 |
| 6 | 11 | 13 | 0 | 0 |
| 7 | 12 | 6 | 9 | 19 |
| 8 | 9 | 10 | 6 | 11 |
| 9 | 9 | 8 | 0 | 3 |
| 10 | 5 | 3 | 0 | 0 |
| 11 | 11 | 15 | 0 | 0 |

Results Rutter A, Rutter B Questionnaires
Sibling scores
Key:
A/B total score at diagnosis
A1/B1 total score at follow up

The results indicate that 2 siblings showed an increase in behavioural disturbance (i.e. increase score of +3 or more) as perceived by parents. In contrast 3 siblings showed a decrease in behavioural disturbance (i.e. decrease score −3 or more) as perceived by parents. The results of the teachers' questionnaire (Rutter B) indicate 3 siblings showed an increase in behavioural disturbance, while 3 siblings showed a decrease in behavioural disturbance (i.e. a change of score of + or −3).

These findings would support the findings of previous studies demonstrating the adverse effects on siblings in the family where a child has leukaemia, and illustrate that distrubance may manifest itself both at time of diagnosis or during the follow up period.

It is also of interest that behavioural disturbance as perceived by the parents may not be reflected in behaviour in the school, and vice versa. It is possible that a child in the home situation may learn to control behaviour for the sake of the family while feeling more free to express their distress in the school context.

The results of the Rutter A and Rutter B questionnaires in 10 patients are presented in Table 2. Rutter B scores are presented on 3 children, the remainder were pre-school children.

The results indicate that 6 patients showed a significant increase in behavioural disturbance (i.e. increase score of +3 or more), as perceived by parents. It is of particular interest that the pre-school children showed a marked increase in behavioural distrubance at follow up.

**Table 2**

**Identified patient scores**

| I.P. | A | A1 | B | B1 |
|---|---|---|---|---|
| 1 | 10 | 20 | Pre-school | |
| 2 | 6 | 9 | 9 | 8 |
| 3 | 9 | 11 | 4 | 7 |
| 4 | 11 | 11 | 2 | 0 |
| 5 | 4 | 9 | pre-school | |
| 6 | 0 | 14 | " | " |
| 7 | 8 | 10 | " | " |
| 8 | 12 | 22 | " | " |
| 9 | 10 | 6 | " | " |
| 10 | 8 | 17 | " | " |

Key:
A/B total score at diagnosis, A1/B1 total score at follow up

Results Rutter A, Rutter B, Questionnaires
Identified Patient Scores (I.P.)

### KINETIC FAMILY DRAWINGS (KFDs)

KFDs were scored using the method described by Spinetta (1981). Drawings were scored on 19 items giving a total possible score of 35. Three subscales were identified to assess aspects of communication, self-image and emotional tone. Drawings were obtained at diagnosis and at one-year follow up.

Table 3 shows results from an incomplete number of KFDs. These preliminary findings appear to show some significant trends. Previous studies have indicated the vulnerability to emotional distress of siblings within the leukaemic family. The KFDs studied so far appear to support this notion. Examination of the KFDs completed by mothers shows a trend towards lower total scores at one-year follow up suggesting a degree of adjustment and adaptation over time. In contrast to this finding it would appear that in the case of fathers the reverse is true. Our preliminary findings indicate that there is a trend for fathers to score higher at one-year follow up, suggesting a reduction in adjustment and adaptation.

### DISCUSSION

It is not possible to draw definitive conclusions from our preliminary findings. It would appear however that the

**Table 3.**

**Kinetic family drawings—siblings/identified patient**

| SIB | TD | TF | I.P. | TD | TF |
|---|---|---|---|---|---|
| 1 | — | 11 | 1 | 10 | — |
| 2 | 8 | — | 2 | 14 | 10 |
| 3 | 15 | 16 | 3 | 17 | — |
| 4 | 7 | 12 | 4 | 14 | 15 |
| 5 | 12 | 17 | 5 | 12 | 12 |
| 6 | 11 | 16 | 6 | 3 | 6 |
| 7 | 16 | — | | | |
| 8 | 12 | — | | | |
| 9 | 14 | 9 | | | |
| 10 | 7 | 8 | | | |
| 11 | 7 | 12 | | | |
| 12 | 15 | 14 | | | |
| 13 | 11 | 14 | | | |
| 14 | 7 | 6 | | | |

Children aged 6+ yrs completed KFD's

Key: TD Total score at diagnosis, TF Total score at follow up

**Table 4.**

**Kinetic family drawings—parents**

| MOTHER | TD | TF | FATHER | TD | TF |
|---|---|---|---|---|---|
| 1 | — | 7 | 1 | — | 11 |
| 2 | 7 | — | 2 | 11 | — |
| 3 | 12 | 9 | 3 | 11 | 16 |
| 4 | 4 | — | 4 | 14 | — |
| 5 | 14 | — | 5 | 6 | — |
| 6 | 13 | — | 6 | — | — |
| 7 | 14 | 8 | 7 | 9 | 21 |
| 8 | 14 | — | 8 | 17 | 9 |
| 9 | 5 | 11 | 9 | 10 | 11 |
| 10 | 11 | 8 | 10 | 12 | 16 |
| 11 | 12 | 10 | 11 | 10 | 15 |
| 12 | 16 | 8 | 2 | 12 | 19 |
| 13 | 16 | 13 | 3 | — | — |
| 14 | 7 | 10 | 14 | 9 | 10 |

Key: TD total score at diagnosis, TF total score at follow up behavioural characteristics of pre-school patients warrant further study. In association with this the parental management style of pre-school patients could warrant further investigation.

The vulnerability of both fathers and siblings has been identified in this study. It is possible that mothers are given more support through their role of carer and through direct contact with the medical team, than fathers and siblings who are more isolated from the treatment process. The findings may also indicate that very young children, and fathers who experience communication difficulties may not be available to support offered.

### ACKNOWLEDGEMENTS

The authors would like to thank the medical and nursing staff of the Oncology Department, Bristol Childrens' Hospital for their help in providing information. They would also like to thank the families who were prepared to share so much of themselves at such a stressful time.

### REFERENCES

COMAROFF, J. and MAGUIRE, P. (1981) Ambiguity and the search for meaning; Childhood leukaemia in the modern clinical context. *Social Science and Medicine,* 158, 115–123.

MAGUIRE, D. P. (1983) Psychological and social aspects of childhood malignancy *Annals Nestle, 41(2), 32–43.*

RUTTER, M, TIZARD, J. and WHITMORE, K.(1970) Education, Health and Behaviour. London, Longman.

SPINETTA, J. J. and DEASY-SPINETTA, P., (1981) Living with Childhood Cancer. London: C. V. Mozby Company.

